# Development of holistic vs. featural processing in face recognition

**DOI:** 10.3389/fnhum.2014.00831

**Published:** 2014-10-20

**Authors:** Kazuyo Nakabayashi, Chang Hong Liu

**Affiliations:** ^1^Department of Psychology, University of HullHull, UK; ^2^Department of Psychology, Faculty of Science and Technology, Bournemouth UniversityPoole, UK

**Keywords:** holistic processing, part processing, developmental face recognition, children, encoding switch, holistic interference, part-whole paradigm

## Abstract

According to a classic view developed by Carey and Diamond (1977), young children process faces in a piecemeal fashion before adult-like holistic processing starts to emerge at the age of around 10 years. This is known as the *encoding switch hypothesis*. Since then, a growing body of studies have challenged the theory. This article will provide a critical appraisal of this literature, followed by an analysis of some more recent developments. We will conclude, quite contrary to the classical view, that holistic processing is not only present in early child development, but could even precede the development of part-based processing.

The encoding-switch hypothesis came from a series of experiments carried out by Carey and Diamond (Carey and Diamond, 1977; Diamond and Carey, 1977; Carey et al., 1980). It was a developmental perspective on Yin’s (1969, 1970) finding that face recognition was more adversely affected by inversion than the recognition of non-face stimuli, such as airplanes or snowflakes. Face inversion is commonly known to disrupt the processing of configural information (i.e., the spatial layout of facial features) that is critical for successful face recognition.

Carey and Diamond found an adult-like inversion effect among 10-year-olds. In contrast, recognition performance by 6- and 8-year-olds was poorer than adults only for upright but not for inverted faces. Based on the assumption that inversion disrupts configural processing, Carey and Diamond reasoned that configural processing is only fully developed at around 10 years of age. The lack of an age difference for inverted faces could be due to all ages using a similar piecemeal encoding strategy. The authors suggested that the emergence of the inversion effect at the age of 10 might reflect the development of the ability to represent upright faces using configural information.

However, a number of subsequent studies have challenged the methodologies and theoretical arguments in Carey and Diamond’s earlier studies. For instance, Flin (1985) argued that young children’s sensitivity to facial orientation could have been masked by a floor effect in Carey and Diamond’s study. If the recognition task is made sufficiently sensitive, age difference in children’s recognition of inverted faces should be observed. To reduce the likelihood of floor effects, Flin re-examined the inversion effect using a small number of faces with long inspection time in an old-new recognition task. As expected, all 7- to 16-year-olds children in the study showed a typical inversion effect.

In fact, there is a growing body of evidence demonstrating that the inversion effect emerges much earlier than previously suggested. Infants’ sensitivity to face orientation has been reported in a number of studies (e.g., Fagan, 1972; Cohen and Cashon, 2001; Turati et al., 2004; Bhatt et al., 2005; Leo and Simion, 2009; Zieber et al., 2013). For instance, 5-month-olds were sensitive to changes to configural information in upright faces, but not in inverted faces (Bhatt et al., 2005). Moreover, 4-month-olds recognized upright faces better than inverted faces when identifying the target face (Turati et al., 2004). Furthermore, even newborns were sensitive to the orientation of a face as their preference for attractive faces disappeared when the faces were inverted (Slater et al., 2000). Newborns, as young as 1- to 3-days-old (Leo and Simion, 2009), exhibited sensitivity to first-order configural information (i.e., the spatial layout of facial features common to all human faces) as they showed a preference for faces as opposed to non-face stimuli of comparable visual complexity (e.g., Johnson and Morton, 1991). These results provide compelling evidence for configural processing at a very early stage of life.

It is worth noting that the presence of the inversion effect at an early age only demonstrates the existence of holistic or configural processing. However, this does not mean that such processing is already adult-like or that it will not go through further development. In addition, as prior studies used different tasks (e.g., preferential looking vs. a standard recognition task) tailored to the age of participants, it would be difficult to assess whether the observed effects across studies reflect identical holistic/configural processing.

Moreover, some researchers have pointed out that featural and configural manipulations may not differ in a fundamental way. For instance, Riesenhuber et al. (2004) hypothesized that the inversion effect on configural processing could have resulted from separating faces with configural and featural transformations into different blocks. Such a design could encourage a specific recognition strategy for detecting one type of change, rather than provoking generic face recognition strategies. As predicted, they found no difference in the recognition of inverted configural and featural changes when they were presented in the same block. Others have also questioned the featural and configural distinction (e.g., Sekuler et al., 2004; Yovel and Duchaine, 2006). It would be interesting to establish whether these results could be replicated in children.

Another piece of key evidence for the encoding switch hypothesis comes from the paraphernalia-to-fool paradigm in which Carey and Diamond (1977) manipulated facial expressions and paraphernalia (e.g., hat, hairstyle, and glasses). Here, children were first presented with a target face. Next the target was paired with a distractor face. The task was to identify which face in the pair was the target. The target varied either in facial expression (no expression or expression) or paraphernalia (paraphernalia removed or added), whereas the distractor either wore the same paraphernalia or posed the same expression as the target during inspection.

The finding was that 6- and 8-year-olds were highly susceptible to errors when the distractor face wore the same paraphernalia as the target. This tendency declined markedly among 10 year-olds. Young children’s reliance on paraphernalia in making identity judgments was also demonstrated when the pair of images were presented simultaneously (Diamond and Carey, 1977). However, when faces were familiar (e.g., classmates), children were able to ignore paraphernalia but used cues that were diagnostic to identity. Diamond and Carey argued that young children represent unfamiliar faces in terms of isolated features when making identity judgements. However, children at the age of around 10 years start to show an adult level of capacity for efficient processing of configural information of a face. The authors suggested that the developmental changes could be due to increased experience with faces in general, but also it may be a result of the maturation of the right hemisphere for dealing configural representations of faces.

Subsequently, Lundy et al. (2001) suggested that the inconsistencies in the literature could be explained by stimulus size. Studies reporting holistic processing in young children used small images (e.g., Flin, 1985; Baenninger, 1994) while others arguing for a change from part to whole processing used larger images (e.g., Schwarzer, 2000). Young children have a tendency to process a stimulus as an undifferentiated whole when the overall image can be perceived in a single glance. Moreover, young children’s differentiation of stimulus components may be related to a limitation in the ability to narrow the focus of attention (see Enns and Girgus, 1985). Hence, the influence of paraphernalia is likely to vary depending on stimulus size as well as age related attentional limitations.

Based on these considerations, Lundy et al. conducted a paraphernalia-to-fool study to examine the effects of visual angle size on face recognition across 3-, 7-, and 10-year-olds. As predicted, 10-year-olds performed better than the two younger groups who showed equivalent performance. The size of a visual angle influenced only 7-year-olds’ performance, with a significant improvement with a large visual angle. However, unlike Diamond and Carey, Lundy et al., did not treat the children’s susceptibility to paraphernalia as evidence for piecemeal processing. Rather, they interpreted it as evidence for younger children’s tendency to process a small image more holistically. According to this, the younger children in Carey and Diamond’s study treated faces and paraphernalia as undifferentiated wholes whereby paraphernalia is processed as part of the face. Clearly, this was an important shift from Carey and Diamond’s interpretation of the same effect. Whilst Carey and Diamond considered holistic processing as the ability to separate irrelevant features from relevant facial information, Lundy et al. treated the effects of irrelevant features as evidence for holistic processing.

Although paraphernalia (e.g., a change of a hair style or addition of glasses) can also affect adults’ face recognition, it does not directly influence their configural or featural processing (Righi et al., 2012). It is because adults tend to include paraphernalia as part of a face, which disrupts encoding of relevant holistic information. Consistent with Lundy et al., Freire and Lee’s (2001) paraphernalia study found that children have the capacity to process configural information by 4 years of age. Nevertheless, misleading paraphernalia could still hamper recognition as children’s memory is susceptible to superfluous information. Therefore, distracting effects of paraphernalia do not necessarily provide evidence for a lack of configural processing as they could be due to a limitation in more general cognitive abilities.

Moreover, as Baenninger (1994) pointed out, the results from Carey and Diamond’s study could have been derived from the distinctiveness of paraphernalia that distracted children’s attention away from relevant information of a face. Furthermore, Flin (1985) argues that distinctive paraphernalia would be of greater perceptual salience than relevant facial information when target-distractor similarity is high, making children more susceptible to distracting effects of paraphernalia. Flin showed that when facial information was made salient (target-distractor dissimilar), 6-year-olds accurately judged the identity while ignoring paraphernalia. However, when target-distractor similarly was high, 4- and 6-year-olds made their identify judgements based on paraphernalia. Flin, therefore, suggests that Diamond and Carey’s (1977) findings could be explained by the task difficulty in that young children ignored relevant facial information while attending only to salient paraphernalia cues. Subsequently, Carey and Diamond (1994) modified their original encoding hypothesis by stating that even young children process faces holistically.

Studies using the composite-face paradigm also support holistic processing among young children (e.g., Tanaka et al., 1998; Pellicano and Rhodes, 2003; see also Carey and Diamond, 1994; Carey, 1996; for a modification of their original theory). The composite effect was first reported by Young et al. (1987), who studied how adult face recognition could be affected when the top half of a face was combined with the bottom half of another face. Typically, participants had a greater difficulty with identifying the top half of the composite face when the two halves were aligned than misaligned. This provided strong evidence that adults tend to perceive the composite face as an undifferentiated whole (see Rossion, 2013 for recent review of the composite effect).

The composite effect has later been demonstrated among 6-year-olds (Carey and Diamond, 1994) and preschool children (de Heering et al., 2007; Cassia et al., 2009). Like adults, children’s identification of the top part of a composite face was better when the face was misaligned than aligned. In fact, Susilo et al. (2009) even reported a lager composite effect for 8- to 13-year-olds than adults when the stimuli were child faces. Subsequently, Turati et al. (2010) showed that infants as young as 3-month-olds exhibit the composite effect, indicating that they are capable of processing faces holistically.

Overall the literature appears to show no fundamental difference in the way children and adults use holistic information to perceive, store, and recognize faces (e.g., Tanaka et al., 1998; de Heering et al., 2007; Mondlock et al., 2007). This leaves an unanswered question: if holistic processing does not separate children’s face processing from that of adults, then what might explain the difference in their recognition performance. We suggest that the answer may partly lie in the inverse of the original encoding switch hypothesis. That is, it may be the proficiency of piecemeal, rather than holistic, processing that takes longer to develop. The evidence for this comes mainly from a holistic interference effect on facial part recognition.

The holistic interference effect was studied in a variant of the part-whole paradigm first developed by Tanaka et al. (Tanaka and Farah, 1993; Tanaka and Sengco, 1997). Unlike the earlier paraphernalia studies that examined the effects of an addition or elimination of non-facial cues (e.g., glasses or hat) which are not part of facial components on identify judgments, the part-whole paradigm directly measures holistic vs. featural processing by examining part recognition in or out of the facial context. Tanaka et al. discovered that adults’ part recognition was better in a whole face than in isolation. In a subsequent study, Tanaka et al. showed that by 6 years of age, children displayed similar holistic effects as adults (Tanaka et al., 1998). Hence children’s face processing does not change from a part to holistic based strategy from this age. Pellicano and Rhodes (2003) later extended the finding to 4-year-olds by showing that children at this age remembered face parts better when tested in a whole face than in isolation.

Leder and Carbon (2005) subsequently provided a fresh approach to the part-whole paradigm using adults. They pointed out that empirical evidence for the whole face advantage had emerged when whole faces were learnt, leaving the gap in our knowledge as to whether the same advantage would still arise when facial parts are learned without the context of a whole face. An underlying assumption was that if learning parts imposes a strict part-based representation, an additional context at test might be ignored. Hence, performance in whole and part conditions may be comparable.

However, the authors found that when isolated parts were learned, presenting the parts in a whole face at test impaired part recognition. This finding was also seen even when participants knew which a critical part was, which implies that it was the unexpected context that hindered part recognition, but not uncertainly about the critical part *per se*. These results suggest that the interaction between facial features and the whole plays a key role in adult face processing. Leder and Carbon argue that the holistic interference is an essence of holistic processing because it demonstrates how difficult it is to ignore irrelevant facial information in a whole face.

Nakabayashi and Liu (2013) have subsequently investigated the holistic interference effect among children. They found that the effect was strongest among the 6-year-olds relative to 9–10-year-olds or adults. Participants in their study judged whether a sequentially presented pair of probe and target eyes were of the same child in four conditions: (1) both probe and test were isolated eyes (part-part); (2) probe was isolated eyes but tested in a whole face (part-whole); (3) probe was a whole face and tested with a part (whole-part); (4) both probe and test eyes were presented in a whole face (whole-whole). The results showed developmental differences when a part was presented in a whole face (i.e., part-whole, whole-part, and whole-whole), with 6-year-olds showing poorer part recognition performance than 9–10-year-olds or adults. In the part-part condition, 6-year-olds were able to identify the parts as well as the two older groups. These findings suggest that holistic processing is already present at 6 years of age. More importantly, the results demonstrate that it is the ability to inhibit the influence of this holistic processing on part recognition that seems to require a longer period of development.

Nakabayashi and Liu suggest that the developmental differences in part recognition may reflect differences in general inhibitory abilities. For instance, research using the go/no-go task procedure reveals that children’s ability to inhibit an automatic response continues to develop throughout childhood (e.g., Dowsett and Livesey, 2000; Brocki and Bohlin, 2004; Berwid et al., 2005). The go/no go task typically requires a key-press response to frequently presented “go” stimuli while inhibiting responses to “no-go” stimuli. Nine-year-olds exhibited better inhibitory processes to soccer balls than 7-year-olds (Cragg et al., 2009). As children get older, they become more able to inhibit their responses at an earlier stage of responding. Perhaps the lack of maturity in this ability among 6-year-olds in Nakabayashi and Liu’s study led to their susceptibility to holistic interference. Their 6-year-olds were less able to inhibit the tendency to process irrelevant information as part of a face.

Evidence for a slower development of piecemeal, as opposed to holistic, processing can also be found in a study by Liu et al. (2013), who investigated the development of facial feature processing in 8–9-, 13–14-year-olds, and adults. In one experiment, participants learnt whole faces, followed by a standard old-new recognition test whereby they identified one of the following test items: eyes; nose; mouth; inner face; outer face; or whole face. The results showed no age difference in the recognition of whole faces, but unlike adults and 13–14-year-olds, 8–9-year-olds were unable to distinguish between old and new facial features. More importantly, when part recognition was preceded by whole learning even 13–14-year-olds did not seem to naturally encode and recognize isolated parts. Based on these findings, the authors suggest that the processing of isolated facial regions differs in its developmental course from that of holistic processing, and that holistic processing may be more dominant before adulthood relative to featural and configural processing.

Results reported in these studies call for a more radical revision of the classical encoding switch hypothesis. The reverse of it may be a more accurate description of the developmental trajectory. It seems that holistic processing is a default mode of processing from an early age as there appears no qualitative difference in the way young children and adults use holistic information. The real developmental differences seem to lie in the ability to successfully encode and extract a critical part from a whole face while ignoring irrelevant information. It is this successful execution of piecemeal processing that seems to take longer to develop to an adult level.

Finally, we should caution that although this review has focused on the development of face recognition, we are not making any assumption that the developmental pattern is specific to face perception. As few studies have made a direct comparison between face and non-face visual processing in the developmental literature, it would be difficult to ascertain whether the development of face recognition has its own unique course. However, some researchers have examined whether certain mechanisms are unique to faces by using both faces and non-face objects (e.g., Yovel and Duchaine, 2006). This line of enquiry will be important for future research because it addresses the question of whether the current knowledge about face recognition development is domain specific or domain general.

## Conflict of interest statement

The authors declare that the research was conducted in the absence of any commercial or financial relationships that could be construed as a potential conflict of interest.
